# ShAn: An easy-to-use tool for interactive and integrated variant annotation

**DOI:** 10.1371/journal.pone.0235669

**Published:** 2020-07-07

**Authors:** Venkat Subramaniam Rathinakannan, Hannu-Pekka Schukov, Samuel Heron, Johanna Schleutker, Csilla Sipeky

**Affiliations:** 1 Institute of Biomedicine, University of Turku, Turku, Finland; 2 Department of Medical Genetics,Genomics, Division of Laboratory, Turku University Hospital, Turku, Finland; CNR, ITALY

## Abstract

**Motivation:**

Annotation of large amounts of generated sequencing data is a demanding task. Most of the currently available robust annotation tools, like ANNOVAR, are command-line based tools which require a certain degree of programming skills. User-friendly tools for variant annotation of sequencing data with graphical interface are under-represented.

**Results:**

We have developed an interactive application, which harnesses the easy usability of R Shiny and combines it with the versatile annotation features of ANNOVAR. This application is easy to use and gives comprehensive annotations for user supplied vcf files using multiples databases. The output table contains the list of variants and their corresponding annotation presented within the graphical interface. In addition, the annotation results are downloadable as text file.

## Introduction

In the era of genomic medicine, next generation sequencing technologies are generating large amounts of human genomics data. There is therefore a great need for an informatics-based analysis method to annotate novel single nucleotide polymorphisms (SNPs) and identify the functionally important variants. ANNOVAR (Annotate Variation) was developed exactly for this purpose [1].

ANNOVAR also includes other functionalities, for example the ability to perform gene-based annotation, region-based annotation and filter-based annotation. It downloads the latest annotation databases and saves them to a local disk where it uses them for the annotation of supplied input files. ANNOVAR is a command line driven Perl based tool, which can run on diverse systems. This tool is inherently complicated for researchers without programming backgrounds to use.

To enable researchers without a programming background to interactively annotate their data, we have designed a new R shiny based interactive application called ShAn (Shiny Annotation) [2] ⁠. The application can either be run locally on a personal computer or uploaded to a server to be used as a web-based application. The user interface created in the application makes the usability of ANNOVAR simpler and easier for researchers without programming knowledge.

## Design and development

The user interface of ShAn was made using libraries available in R. The interactive data is presented in the main panel and the options for uploading and annotating the data is given in the collapsible side panel. The user can choose between two types of database selection for the annotation: “gene” and “filter”. By selecting either of these options after uploading the input vcf file the user is able to interactively select one or more databases to use for the annotation.

When choosing “gene”, the user is able to make an annotation based on four databases. However, when choosing the “filter” option a larger number of seven databases are available. On the user interface the user can also select the human genome version as reference for the annotation. Presently genome versions Hg19 and Hg38 are supported. At this stage, the user is able to view an additional selection called the “FAQ”. This contains frequently asked questions and basic troubleshooting advice for the application.

In more detail, selecting the “gene” option gives the ability to do gene-based annotation. The databases that can be used for annotation are represented in abbreviated form in the tool:

- RefGene: Consists of human protein coding genes taken from the National Center for Biotechnology Information RNA reference sequences collection.
- KnownGene: The knownGene database is based on the University of California Santa Cruz Genome Browser knownGene track. KnownGene shows gene predictions based on data from RefSeq, Genbank, Consensus coding sequence and the Universal Protein Database.
- EnsGene: Ensembl genome database is a data source jointly maintained by the European Bioinformatics Institute and the Wellcome Trust Sanger Institute.

The “filter” option initiates a filter-based annotation with the selected database. Here, only the exact variants with the same start and end positions, and with the same observed alleles, are annotated. The abbreviations used in the tool and a brief description of the databases are as follows:

- 1000g2015aug: The largest public catalogue of human variation and genotype data of different populations.
- avsnp150: A reformatted dbSNP dataset for use in the ANNOVAR tool. The indels are left normalized and there is just one variant per line.
- esp6500siv2_all: An exonic region database derived from a National Heart, Lung and Blood Institute funded exome sequencing project.
- exac03: The Exome Aggregation Consortium database is an aggregate of exome sequencing data from a wide variety of large scale sequencing projects.
- gerp++gt2: The Genomic Evolutionary Rate Profiling database is used to identify constrained elements in multiple alignments. This database is unavailable for Hg38.
- Cg46: Complete Genomics database is useful for filtering out artifacts in the Complete Genomics platform.
- Dbnsfp35a: This is a database for functional prediction and annotation of all potential non-synonymous single nucleotide variant.
- gnomad211_genome: The Genome Aggregation Database is an aggregate of genome sequencing data from a multiple sequencing projects.

**Fig 1:**
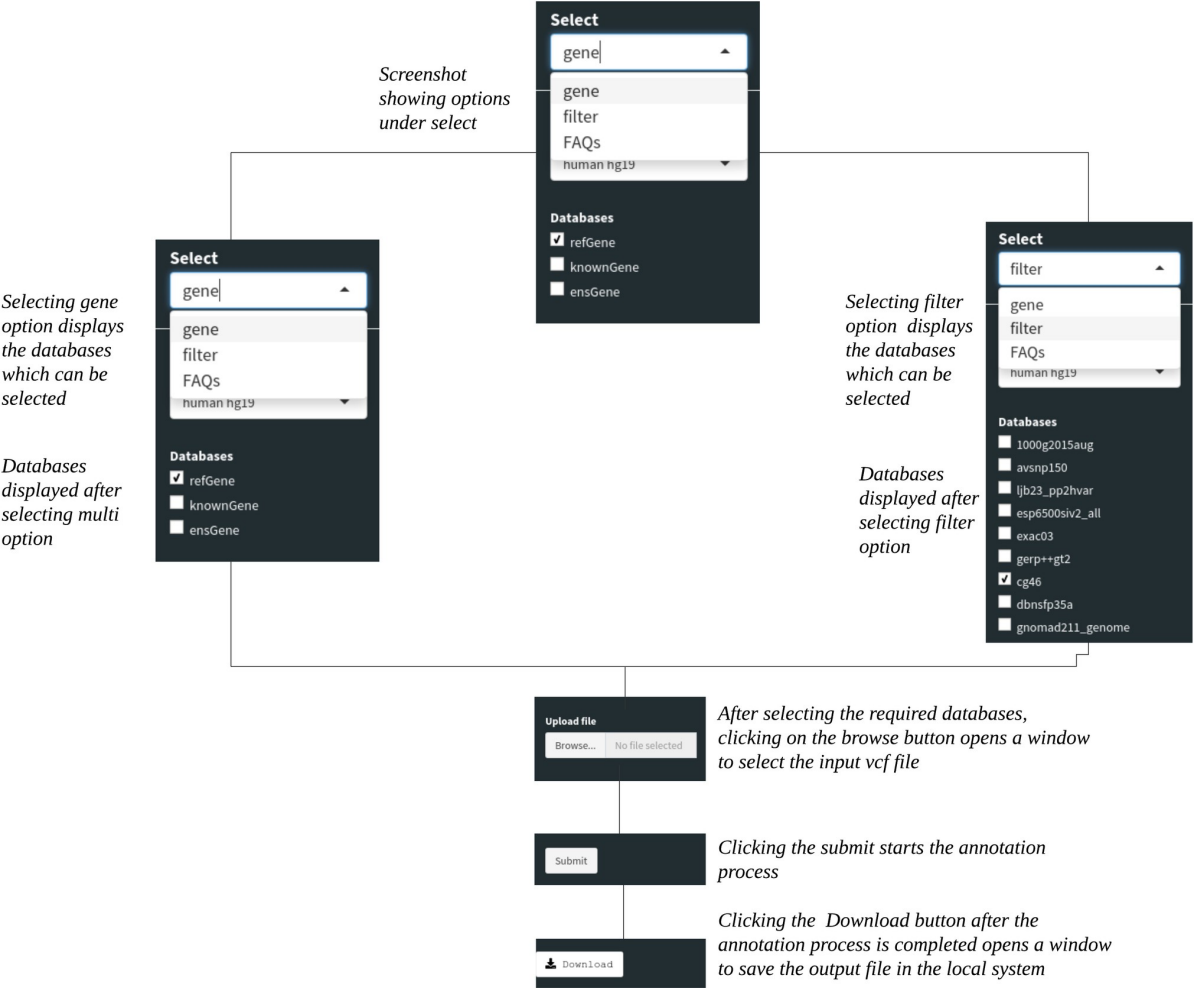
Example usage of the ShAn user interface for each step involved in the annotation process.

The desired annotation databases are downloaded locally and a copy of it is kept in the local system. The output is stored as a temporary file and discarded after each new upload. This was done to reduce the space used in the local system. If the user needs the results file to be saved locally, it can be downloaded as a text file after annotation. Fig 1 shows in brief the user interaction required to start annotation with different options available in our tool.

## Data acquisition and processing

The tool was validated using three datasets; an Illumina gold standard dataset named NA12878, the Ashkenazim trio dataset and an in-house dataset named PrCa_sample.vcf. All three datasets can be found on the tool’s GitLab repository (**https://gitlab.utu.fi/vesura/ShAn.git**).

The NA12878 file is commonly used as a benchmark dataset [3] and this can be downloaded from the Illumina website (https://www.illumina.com/platinumgenomes.html). This is a set of high confidence variant calls that take into account the inherent constraints in the pedigree and the concordance of variant calls across different methods. This is provided in the GitLab repository under the name “NA12878.vcf”.

The Ashkenazim dataset is part of the collection of gold standard variant calls maintained by Genome in a Bottle (ftp://ftp-trace.ncbi.nlm.nih.gov/giab/ftp/release/AshkenazimTrio/). This consists of a son-father-mother trio and the latest vcf files from each of the individuals were used for testing the tool. These are provided in the repository under the folder “Ashkenazim_trio”.

In order to be able to validate ShAn in comparison to the existing graphical interface-based tools Seattleseq and wANNOVAR we created a small in-house prostate cancer vcf file from randomised real prostate cancer data. This is named “PrCa_sample.vcf” and provided in the repository.

## Results and discussion

The features of ShAn were compared to two existing tools: wANNOVAR (web interface to the ANNOVAR software) [4] and Seattleseq. A summary of this comparison is provided in Table 1.

**Table 1:** Advantages of ShAn compared to wANNOVAR and Seattleseq.

	ShAn	WANNOVAR (http://wannovar.wglab.org/)	Seattleseq (https://snp.gs.washington.edu/SeattleSeqAnnotation151/)
**Availability of online hosting**	Yes	Yes	Yes
**Source code accessibility**	Yes (GitLab)	No	No
**Upload file limitations**	There is no file size limitation.	Up to 100 MB	Files containing more than 2,000,000 lines cannot be processed
**Security of data**	Uses own server/personal computer with provided source code	Does not use own server/personal computer. Data to be transferred to international servers	Does not use own server/personal computer. Data to be transferred to international servers
**Display of annotated data**	On screen and downloadable	On screen and downloadable	Only through download
**Tracking Progress**	Progress bar on screen	No progress bar on screen, only email notification	Progress bar on screen and email notification

## Interface, availability and accessibility

ShAn was developed to enable researchers to have easy and instant access to annotation tools, thus aiding in faster progress of their research. ShAn displays the annotated vcf file in a tabular format in the main panel. As described in Table 1 the annotation process is tracked using a progress bar neatly displaying the progress of the annotation at the lower right corner. This enables the user to estimate time of completion. wANNOVAR and Seattleseq use email to notify the user of the completion of the analysis and display the annotated data in the tabular format. As summarized in Table 1, ShAn can be hosted on to a server and made available via the internet. In addition, the source code is made available through a gitlab repository.

## Input file size limitation

In practice, we have found that many vcf files requiring annotation are frequently above the size of 100 MB. Breaking a single vcf file into smaller files makes it harder to analyze the annotated output. Currently, wANNOVAR and Seattleseq both have input file size limitation of 100 MB and 2,000,000 lines per vcf file, respectively. In comparison, ShAn does not impose file size restriction on the user (Table 1).

## Privacy and security of data

Variant files requiring annotation frequently contain sensitive genetic information and this data when exposed to the public domain could be compromised. This transfer of data could also violate data privacy and security laws currently in place in the EU for genetic data (https://gdpr-info.eu/). Sensitive patient data comes under the purview of these rules, and hence our tool offers a significant advantage of in-house annotation without the need for exporting the data to servers outside of the EU. Seattleseq and wANNOVAR require the data to be transferred via the public internet to a server based in the United States of America, whereas ShAn can be hosted on a private computer or server, thus circumventing this limitation (Table 1).

## Validation

The validation of the ShAn tool has been carried out using both gold standard (NA12878) and in-house vcf files (PrCa_sample), which have been annotated with existing tools as well and the output files compared for consistency.

The NA12878 and Ashkenazim trio datasets were annotated both with ShAn and ANNOVAR. Both of the tools gave the exact same annotation as output, the output files from this analysis are provided in the ShAn GitLab repository. Of note, here we used the ANNOVAR command-line tool since both of the datasets were too large to be analyzed by Seattleseq or wANNOVAR graphical interface-based tools. This exemplifies one of the advantages of ShAn.

The annotation of the “PrCa_sample.vcf” in-house dataset with ShAn, Seattleseq and wANNOVAR was successful, resulting in a consistent output. These annotations are also provided in the GitLab repository. Of note, Seattleseq did not provide the annotation for the clinical significance, this is present in the annotation produced by both ShAn and wANNOVAR. The tools used for comparison do not provide runtime speeds, load capacities and operating system information on which they are executed. Therefore, unbiased statistical testing of computational performance could not be carried out.

**Fig 2:**
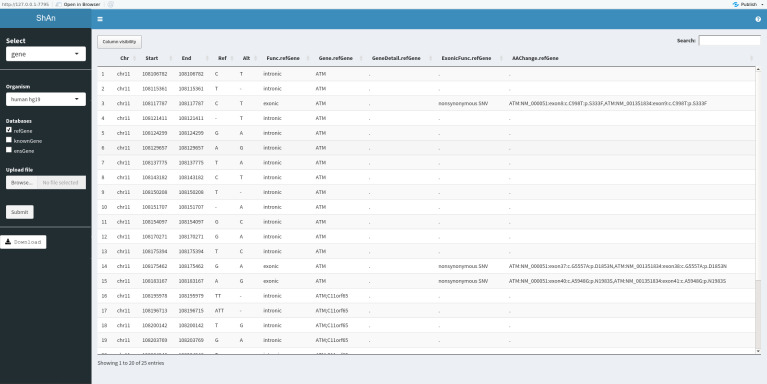
The graphical output of the in-house data variant annotation with ShAn.

A screenshot of the output of the in-house data is provided in Fig 2.

The current version of ShAn is developed for the annotation of SNPs in human data. This version of the tool will be maintained and updated periodically to ensure compatibility with the most recent version of ANNOVAR. The application is planned to be extended in the future through the implementation of support for other organisms, the ability of users to manually update annotation databases and by making new databases available. As ShAn’s source code is modular there is room for further improvements. The application could also be further developed to include pre- and post-processing steps for high throughput data. Additional details for installation and sample annotation is provided in the S1 File.

## Supporting information

S1 File (DOCX)
