# Supplementary material for: ShAn: An easy-to-use tool for interactive and integrated variant annotation
Source: PLoS One. 2020 Jul 7;15(7):e0235669. doi: 10.1371/journal.pone.0235669 (PMC7340278; doi:10.1371/journal.pone.0235669)
Supplement: S1 File — (DOCX) [file pone.0235669.s001.docx]

To run ShAn the system on which it is being executed should have the latest version of R and RStudio. The following steps illustrate how these can be installed if required:

**Step 1: Install R**

1. Install R by visiting <https://cran.r-project.org/> and downloading the installer appropriate for your operating system.


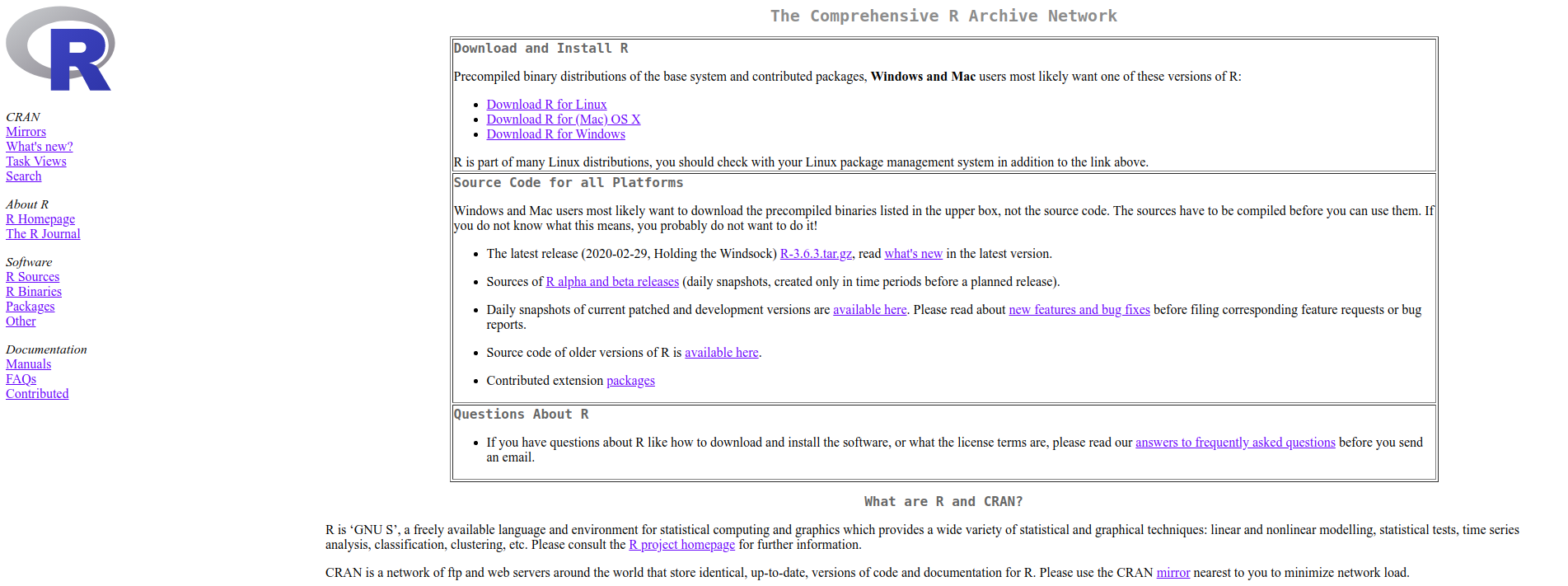
Supplementary Figure 1: The download page on the official R website (<https://cran.r-project.org/>).

2. Run the installer with the default settings. If you don’t have admin rights on your system ask your IT support to provide the required permissions to the R directories. This is important for installing future R packages.

**Step 2: Install RStudio**

1. Download RStudio from <https://rstudio.com/products/rstudio/download/#download> by selecting the installer appropriate for your operating system.


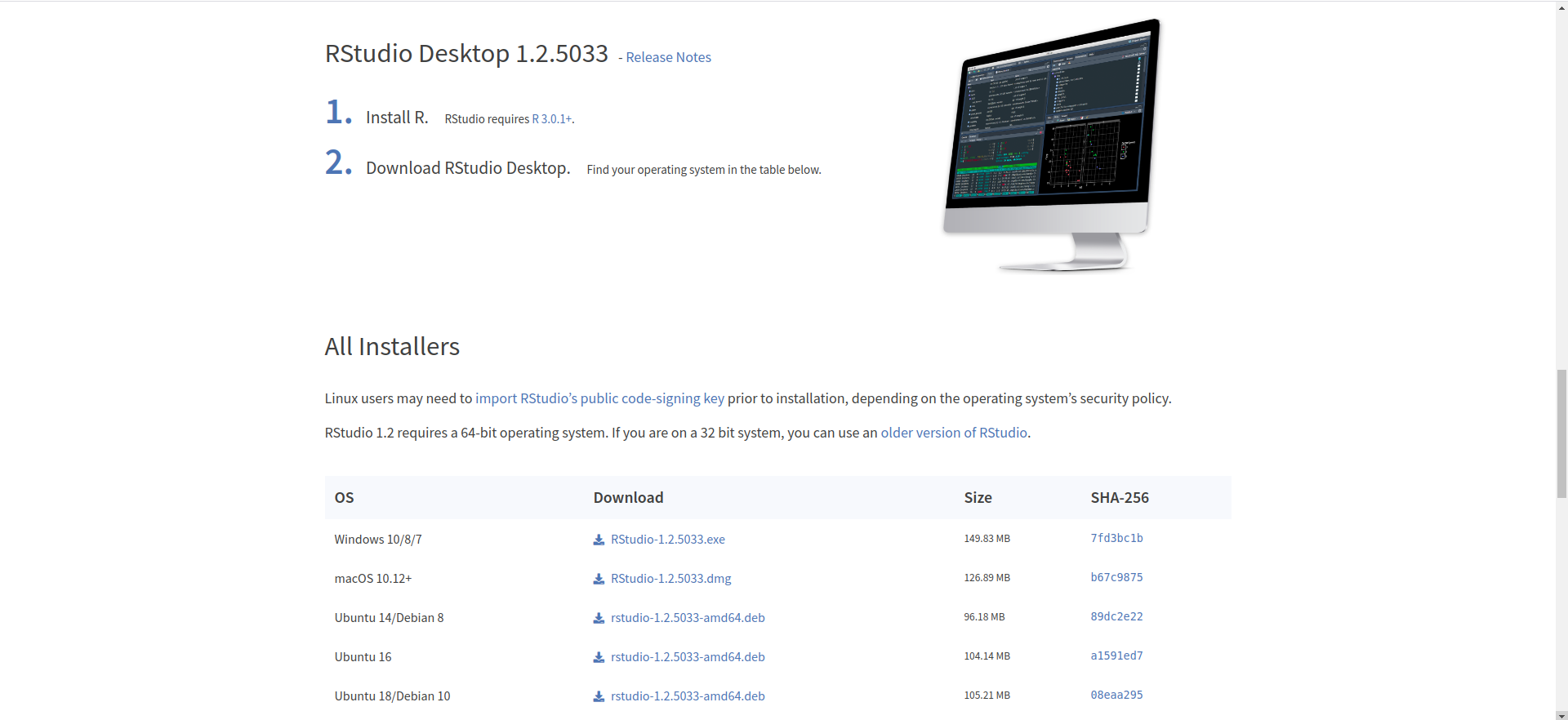
Supplementary Figure 2: The download page on the official RStudio website (<https://rstudio.com/> [products/rstudio/download/#download](https://rstudio.com/products/rstudio/download/#download)).

2. After the installation of R has completed successfully (and not before) run the RStudio installer.

3. Administrative rights need to be granted for RStudio to be installed successfully. If there are problems regarding this please contact your local IT support for the appropriate rights to be granted.

**Step 3: Check that R and RStudio are working.**

1. Open RStudio, the screen should look similar to Supplementary Figure 3 shown below.

2. In left pane beside the ‘>’ symbol, type ‘4+5’ (without quotes) and hit enter. If the output is ‘9’ that means R and RStudio are working. If this isn’t successful please contact your IT support for further assistance.

3. The working directory needs to be set by using the command setwd(“your_file_location”). This is where all the files required for ShAn to function will be saved.


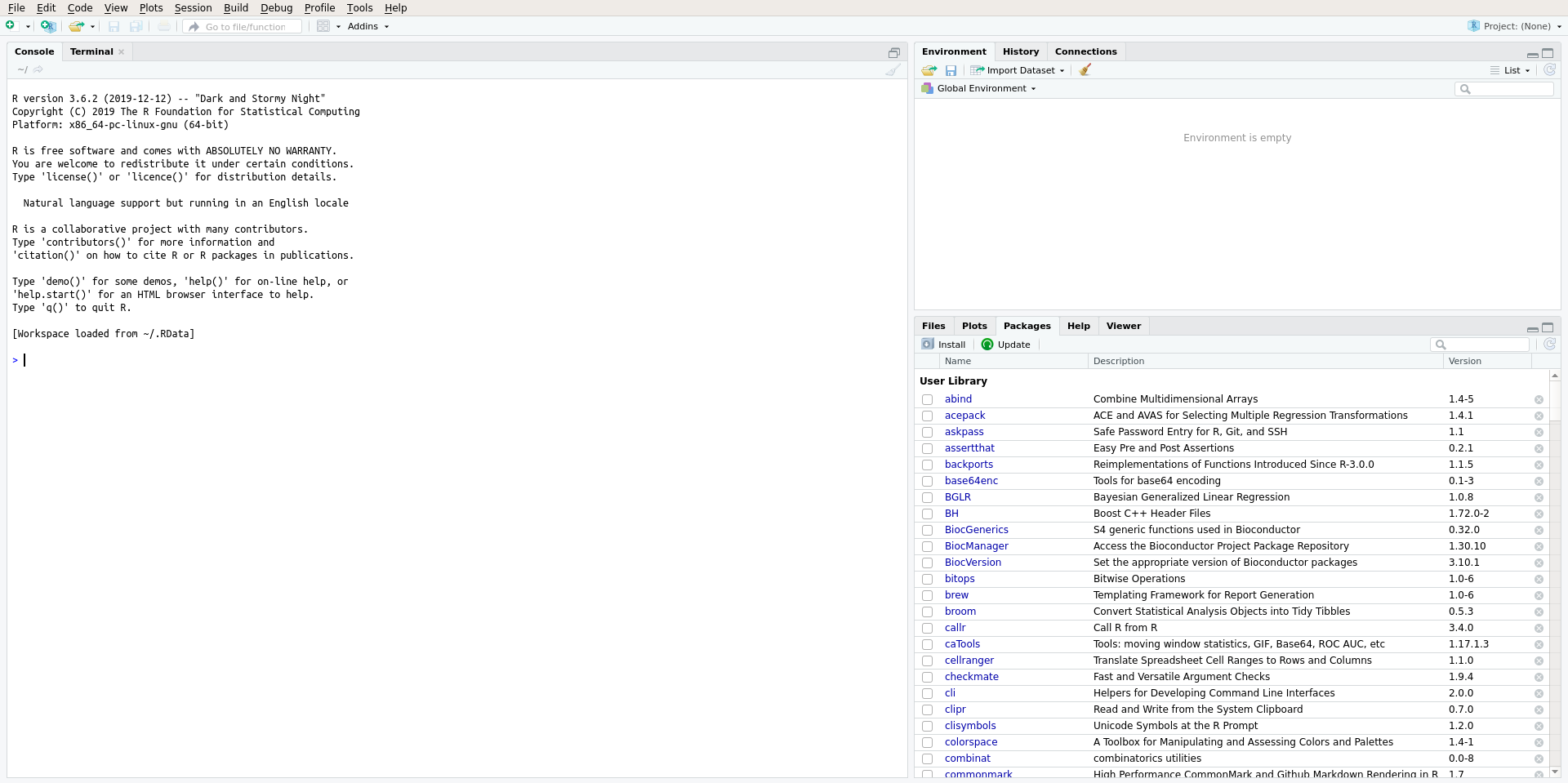
Supplementary Figure 3: The startup page of RStudio running with R.

**Step 4: Installing the required packages**

1. R may ask you to set a mirror to download the packages from and the user should select that which is geographically closest.

2. To run the tool ShAn the following packages need to be installed: shiny, shinyjs, shinydashboard, DT, knitr and rmarkdown.

3. This can be done by typing the following command in the left window as seen in Supplementary Figure 3. The commands are:

install.packages("shiny")

install.packages("shinyjs")

install.packages("shinydashboard")

install.packages("DT")

install.packages("knitr")

install.packages("rmarkdown")

**Step 5: Running the tool**

1. Now download the entire repository from <https://gitlab.utu.fi/vesura/ShAn.git> and extract the files to your system.

2. Open the file named “ui.R” and as seen in Supplementary Figure 4 and click on the “run app” button on the top right hand side of the pane.

3. If the “run app” button does not appear please contact your IT support for further assistance.


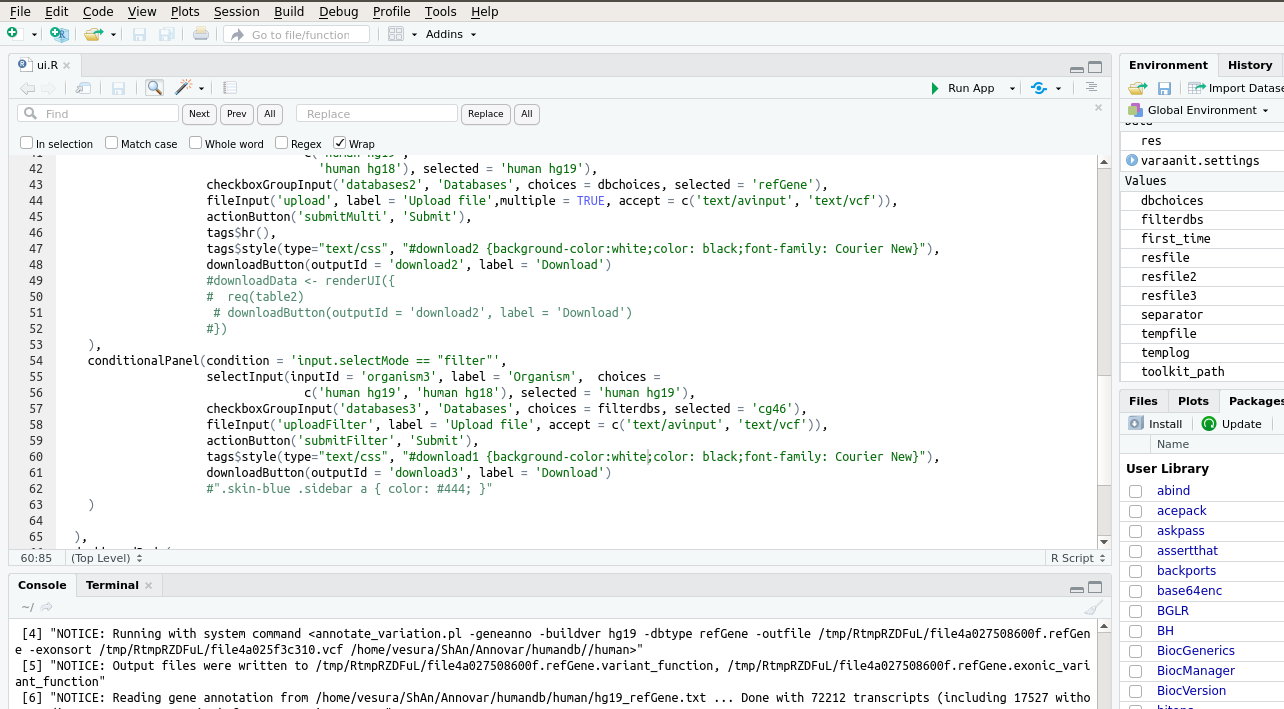
Supplementary Figure 4: Screenshot after opening the ui.R file.

**Step 6: Uploading and annotating a vcf file**

The subsequent steps shows how to upload a vcf file and initiate the process of annotation.

1. After the tool has been run from the RStudio interface, the startup page for the app is shown in Supplementary Figure 5.

Supplementary Figure 5: Screenshot of startup page of ShAn tool.


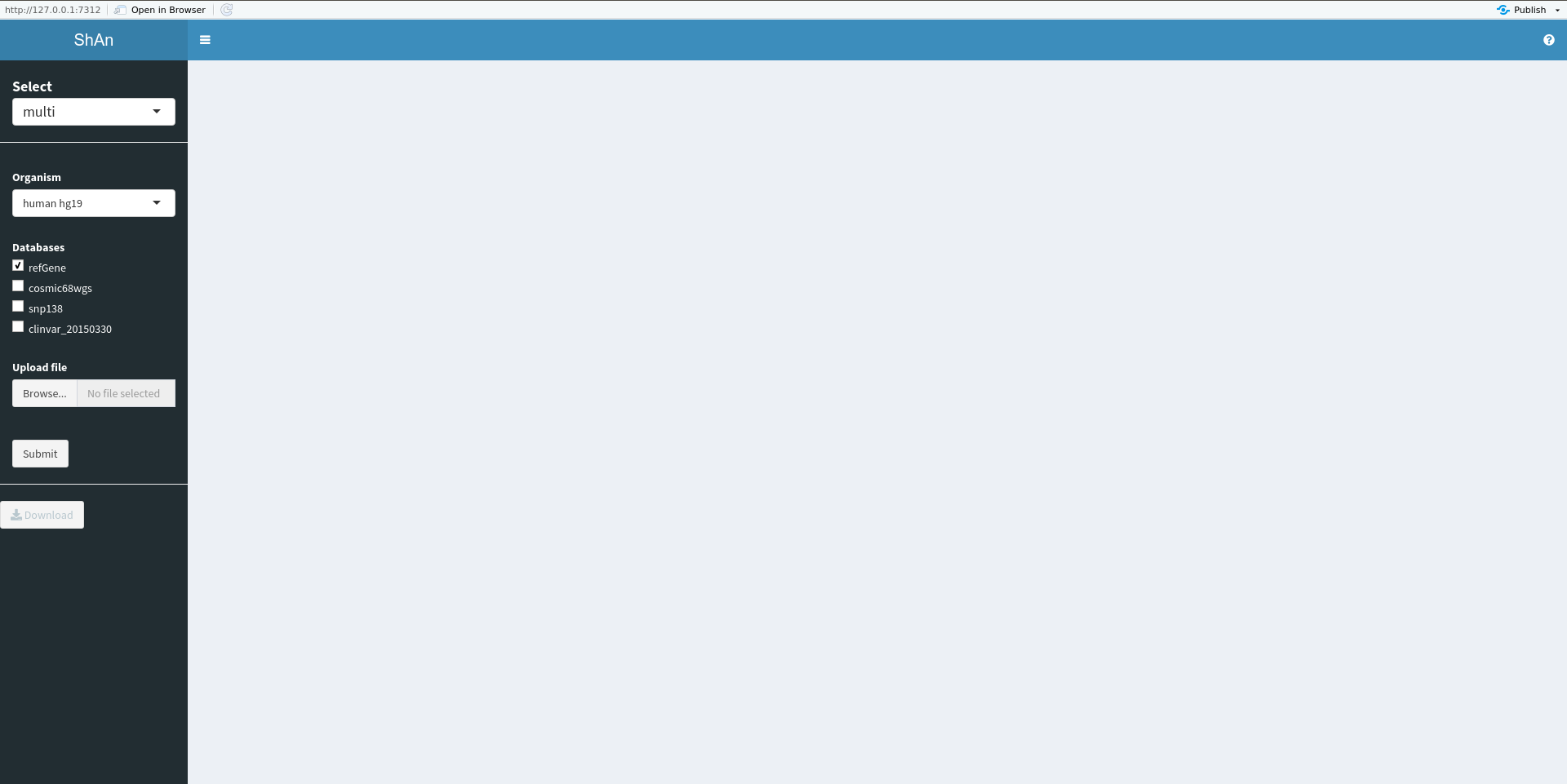


2. Select the annotation type under the “Select” header. The drop down list is shown in Supplementary Figure 6.


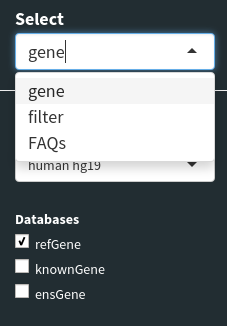


Supplementary Figure 6: Screenshot of options under the “Select” header, gene is selected here and the corresponding databases are shown below.

Selecting the option “gene” gives us three databases to choose from and they can be selected or deselected as required. Selecting the option “filter” gives us a different set of database selection as shown in Supplementary Figure 7.


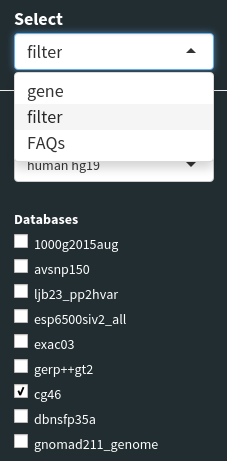


Supplementary Figure 7: Screenshot of options under the “Select” header, filter is selected here and the corresponding databases are shown below.

3. Clicking the “Browse” button displays a window which should be used to select the vcf file of choice and upload for annotation. The file chosen here is “PrCa_sample.vcf” available in the GitLab repository.


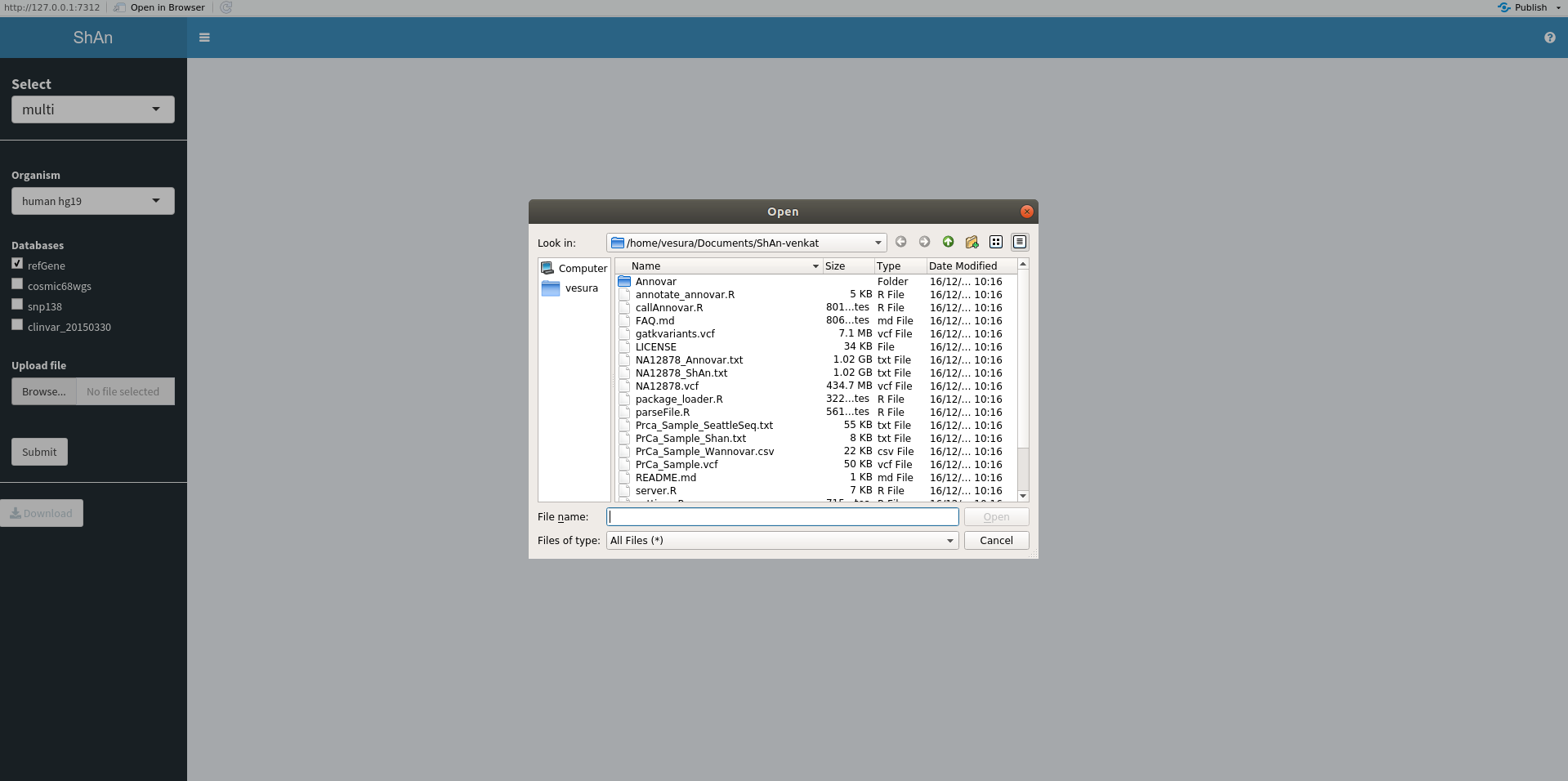


Supplementary Figure 8: Screenshot showing the window to upload the file

4. Once the file has been selected and submit button is clicked the upload process starts and a progress bar is displayed at the right hand corner of the screen as shown in Supplementary Figure 9, this shows the progress of the annotation.


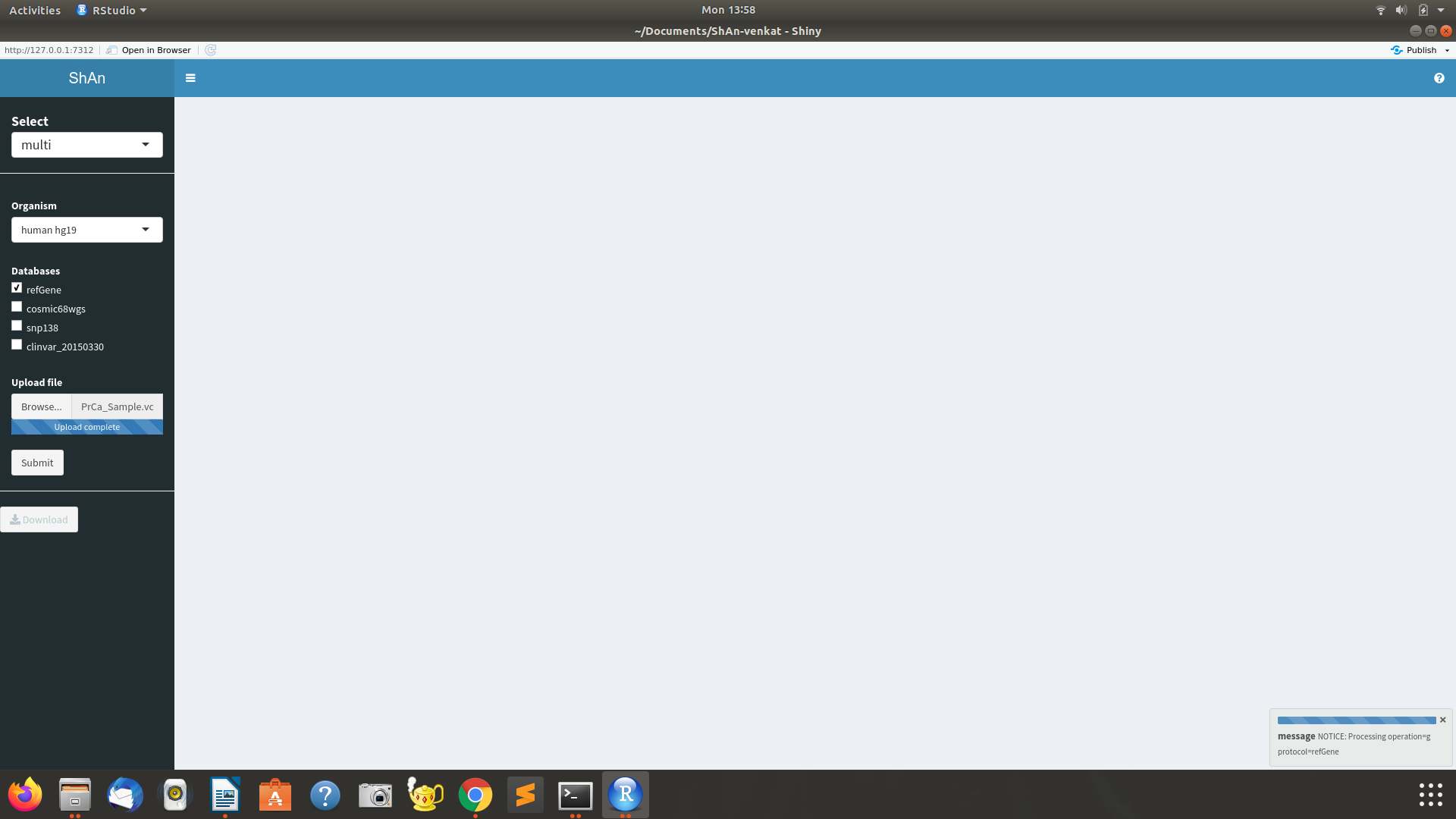


Supplementary Figure 9: Screenshot showing the progress bar in the lower right hand corner.

5. The results for the annotated file are displayed in the right pane as can be seen in Supplementary Figure 8. The result file is also available in the GitLab repository under the name “PrCa_Sample_ShAn.txt”


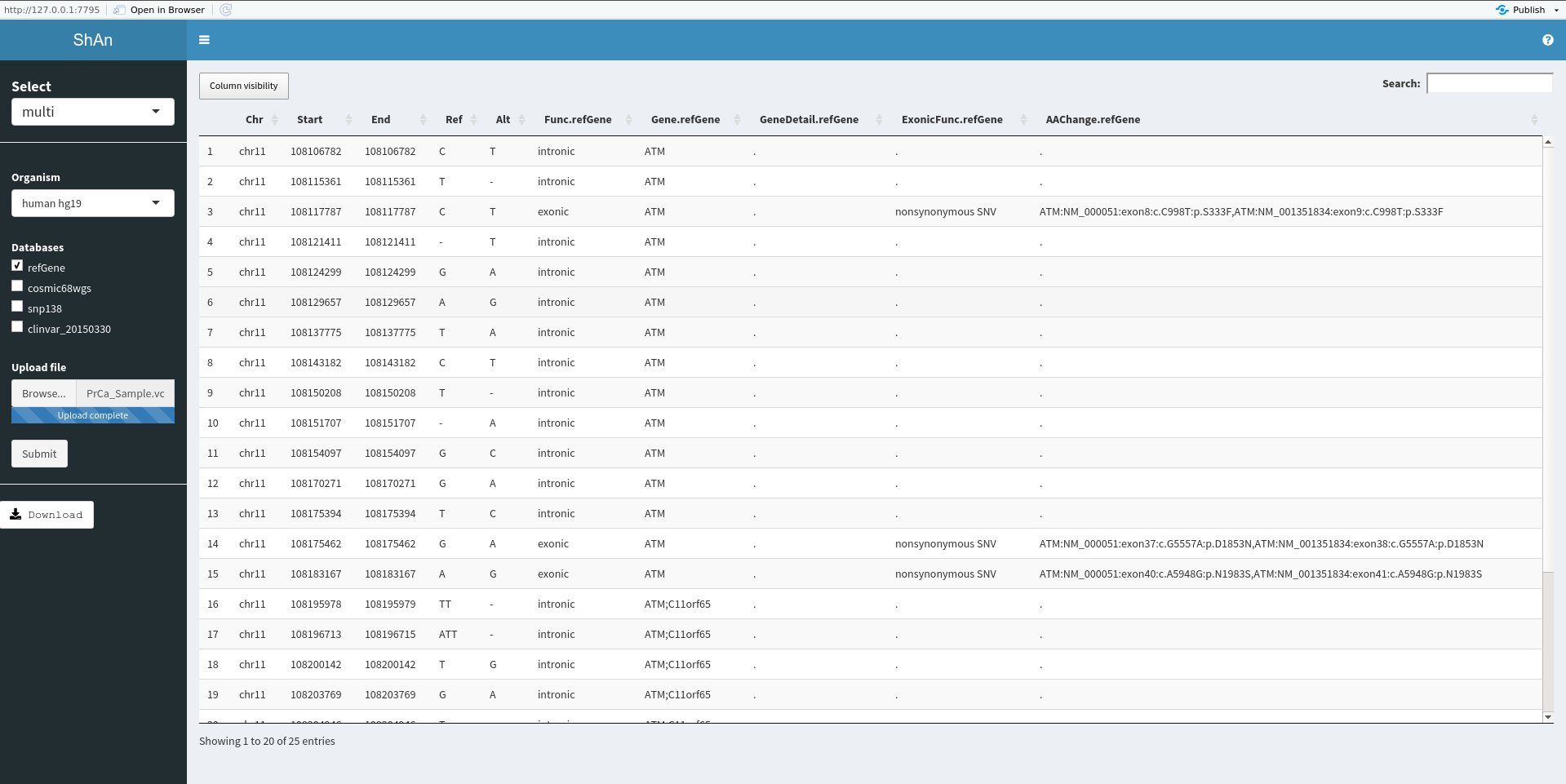


Supplementary Figure 10: Screenshot showing the annotated list on the right pane.

6. The results can also be downloaded by clicking on the download button. This opens a new window where the file can be saved to the users preferred location as seen in Supplementary Figure 11. The file is saved in a .txt format.


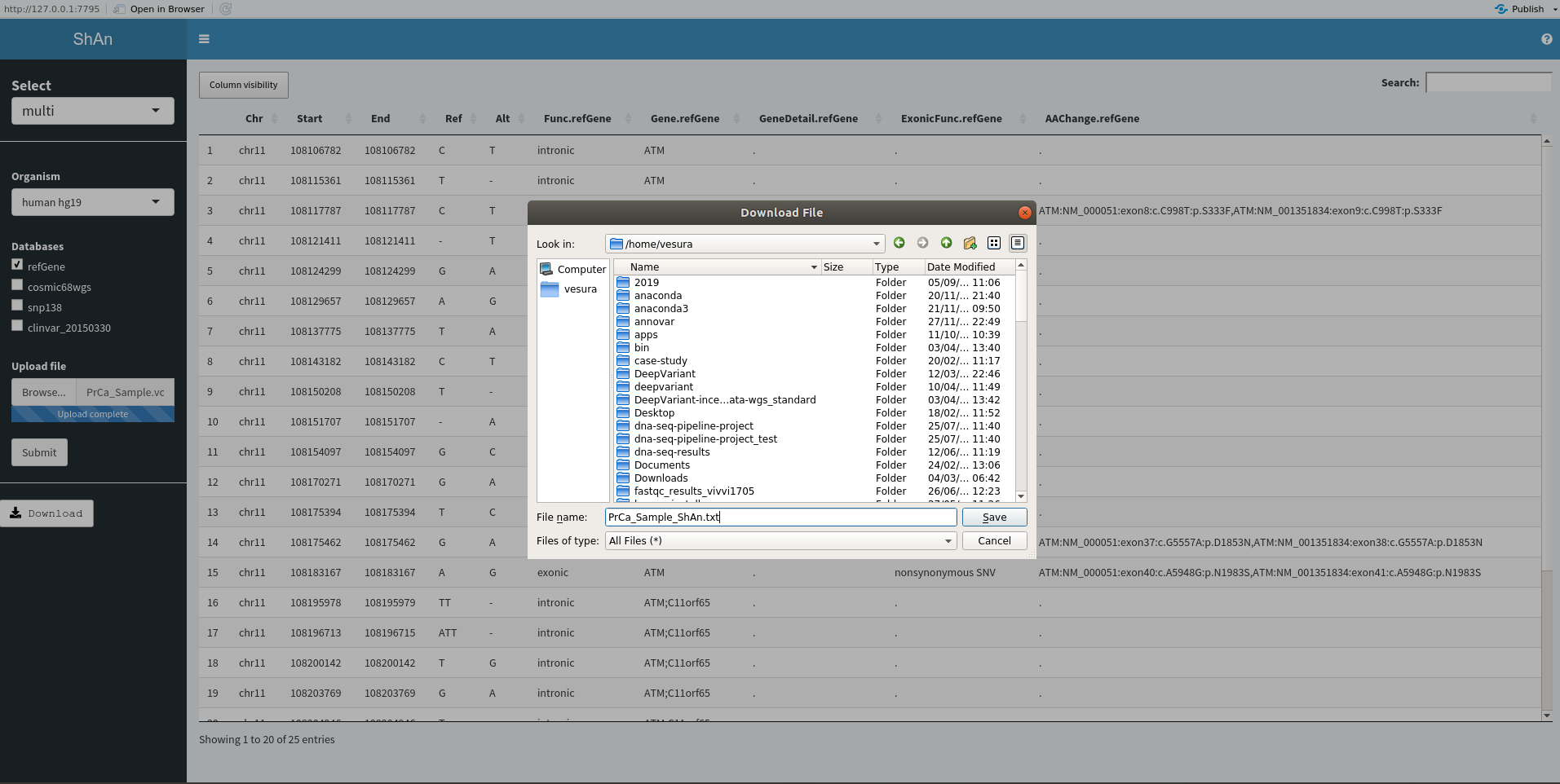


Supplementary Figure 11: Screenshot showing download window to select the location to save the results file in the local system.
